# Antiaflatoxigenic effect of fullerene C_60_ nanoparticles at environmentally plausible concentrations

**DOI:** 10.1186/s13568-018-0544-0

**Published:** 2018-02-05

**Authors:** Tihomir Kovač, Bojan Šarkanj, Tomislav Klapec, Ivana Borišev, Marija Kovač, Ante Nevistić, Ivica Strelec

**Affiliations:** 1 Department of Applied Chemistry and Ecology, Faculty of Food Technology, Josip Juraj Strossmayer University of Osijek, Franje Kuhača 20, 31000 Osijek, Croatia; 2 Department of Chemistry, Biochemistry and Environmental Protection, Faculty of Sciences, University of Novi Sad, Trg Dositeja Obradovića 3, 21000 Novi Sad, Serbia; 3 Inspecto Ltd., Električne centrale 1, 31400 Đakovo, Croatia

**Keywords:** Fullerene C_60_, Nanoparticles, *Aspergillus flavus*, Aflatoxin B1, Oxidative stress

## Abstract

Increased interest in fullerene C_60_ and derivatives in recent years implies an intensification of their environmental spread. Yet, the potential risks for living organisms are largely unknown, including the interaction of C_60_ with fungal organisms. This may be especially relevant for mycotoxigenic fungi since C_60_ may both scavenge and produce reactive oxygen species (ROS), and oxidative stress induces mycotoxin production in fungi. Therefore, this study examined effects of environmentally plausible concentrations of C_60_ (0, 10, 50, and 100 ng/mL) on *Aspergillus flavus* growth and aflatoxin production in culture media. In addition, ROS-dependent oxidative stress biomarkers—thiobarbituric acid reactive substances (TBARS), reduced and oxidised glutathione ratio, superoxide dismutase isoenzymes, catalase, glutathione peroxidase, and glutathione reductase were determined in mycelia. Nanoparticles of fullerene C_60_ (nC_60_) did not exhibit strong antifungal activity against *A. flavus*. At the same time, nC_60_ caused an antiaflatoxigenic effect at 10–100 ng/mL, and 50 ng/mL unexpectedly enhanced aflatoxin production. The TBARS content, reduced and oxidised glutathione ratio, and copper, zinc superoxide dismutase activity suggest that 10 ng/mL nC_60_ exerted antioxidative action and reduced aflatoxin B1 production within fungal cells. Detected prooxidative effects of 50 ng/mL fullerene exceeded cellular defenses and consequently enhanced aflatoxin B1 production. Finally, the results obtained with 100 ng/mL nC_60_ point to prooxidative effects, but the absence of increase in aflatoxin output may indicate additional, presumably cytotoxic effects of nC_60_. Thus, a range of rather low levels of nC_60_ in the environment has a potential to modify aflatoxin production in *A. flavus*. Due to possible implications, further studies should test these results in environmental conditions.

**Electronic supplementary material:**

The online version of this article (10.1186/s13568-018-0544-0) contains supplementary material, which is available to authorized users.

## Introduction

The industry of nanomaterials has started bringing fullerene C_60_ containing commodities to the market, although their commercial applications are still rather modest (Hotze et al. 2008). Recent applications and research of fullerene C_60_ and derivatives include electronics, electrochemical sensors, medicine and cosmetics, textile and food products, etc. (Michalitsch et al. 2008; Duncan 2011; Pycke et al. 2012). Nevertheless, in addition to natural and industrial combustion processes, nanotechnology-related emissions of C_60_ in the environment are expected to rise in the future (Farré et al. 2011; Sanchís et al. 2012, 2013, 2015) despite the alert given by European Commission (European Commission Alert 2012). This underscores the importance of understanding environmental fate and behaviour of C_60_, as well as connected risks. All the potential benefits of fullerene C_60_, which were initially proposed, are shadowed by the possibility of its negative impact on environment and living organisms. For example, the most important environmental forms of C_60_ are stable aggregates mainly present as nano C_60_ or nC_60_ forms in water, were shown to interact with both prokaryotic and eukaryotic organisms (Lyon et al. 2008; Pycke et al. 2012).

Literature data concerning the nC_60_ effect on fungi is limited. Present data suggest that fungi are quite resistant to nC_60_ (Cherepanova et al. 1997; Aoshima et al. 2009; Hadduck et al. 2010). A recent review has similarly concluded that negative effects of carbon nanoparticles on soil microbial communities may be expected only at levels higher than 250 mg/kg (Simonin and Richaume 2015). Caution is, however, required due to differences in studies’ design and/or nC_60_ preparation methods (Lyon and Alvarez 2008; Chae et al. 2010). An obvious knowledge gap exists on fullerene nC_60_ and mycotoxigenic fungi interaction, despite the risks of this interaction ending with either positive or negative outcomes, as outlined below.

The most prominent fullerene properties are its ROS scavenging (Krusic et al. 1991; Gharbi et al. 2005; Trpkovic et al. 2012) and producing ability, with or without photosensitisation (Yamakoshi et al. 2003; Isakovic 2006a; Usenko et al. 2008; Chae et al. 2010). Based on this fact, nC_60_ possesses a great potential for interaction with *Aspergillus flavus*, a mycotoxigenic fungus which is the main producer of aflatoxin B1—the most potent natural carcinogen (Klich 2007; Amaike and Keller 2011; Yu 2012). Oxidative stress induces aflatoxin production in *A. flavus* (Narasaiah et al. 2006; Reverberi et al. 2010; Roze et al. 2013), thus highlighting the nC_60_ potential to modulate aflatoxin synthesis. According to Trpković et al. (2012) low C_60_ doses demonstrate antioxidant effects in contrast to extremely high concentrations, which are highly unlikely in the environment. Sanchis et al. (2015) reported ppq levels (pg/L) of fullerenes C_60_ and C_70_ in wastewaters, surface waters and river sediments while Farré et al. (2010) reported up to 19.1 µg/L of fullerene C_60_ in wastewaters. Sun et al. (2014) predicted annual increase of fullerene in sewage sludge-treated soil between 0.38 and 1.5 µg/kg. Therefore, the aim of present study is to examine the effect of low, environmentally plausible concentrations of nC_60_ water suspensions on *A. flavus* aflatoxin production ability and oxidative stress modulation. Another coincidental emerging issue that increases the relevance of this work revolves around anticipated higher contamination rates of crops with aflatoxins due to climate change (Battilani et al. 2012, 2016).

## Materials and methods

### Chemicals

Superoxide dismutase from bovine erythrocytes (3000 U/mg protein) (SOD), glutathione reductase from baker’s yeast (*Saccharomyces cerevisiae*) (100–300 U/mg protein) (GR), xanthine oxidase from bovine milk (0.4–1.0 U/mg protein) (XOD), xanthine, nitrotetrazolium blue chloride (NBT), ethylenediaminetetraacetic acid tetrasodium salt (EDTA-4Na), l-glutathione, reduced (GSH), l-glutathione oxidised disodium salt (GSSG), potassium cyanide, diethylenetriaminepentaacetic acid (DTPA), *N*-ethylmaleimide (NEM) and sodium dithionite (DT) were purchased by Sigma Aldrich (Germany). β-nicotinamide adenine dinucleotide 2′-phosphate reduced tetrasodium salt (NADPH) was purchased from Serva (Germany), while stabilized 3% solution of hydrogen peroxide (H_2_O_2_), *o*-phthalaldehyde (OPA) and formic acid were purchased from Fluka (Germany). Sodium azide and hydrochloric acid were obtained from Merck (Germany). Aflatoxin standard mix (B1, G1, B2, G2) was purchased from Biopure (Austria). Acetonitrile and methanol (both HPLC grade) were obtained from J. T. Baker (Italy), while yeast extract, potato dextrose agar and sucrose were purchased from Biolife (Italy).

Trichloroacetic acid (TCA) and ascorbic acid were from Kemika (Croatia). Ethylenediaminetetraacetic acid disodium salt 4 (EDTA-2Na) was from Pharmacia Biotech (Sweden), absolute ethanol was from Panreac (Spain), while butylated hydroxytoluene (BHT) and 2-thiobarbituric acid (TBA) were obtained from Acros Organics (USA). Fullerene C_60_ was purchased from Mer Corporation (USA).

### Fullerene C_60_ nanoparticles (nC_60_) solution preparation and characterization

Fullerene C_60_ nanoparticle solution was prepared according to Lyon et al. (2006), with modifications. Briefly, 200 mg of bulk C_60_ was stirred in 500 mL of ultrapure water (Millipore Simplicity 185, Merck, Germany) for 10 min at 550 rpm (Rotamix 550 MMH, Tehtnica, Slovenia) and sonicated (Sonorex Super RK 100 H, Bandelin, Germany) for 60 min in two cycles with 30 min of cooling between cycles. Prepared brown suspension was additionally stirred at 550 rpm for 30 days and then filtered through Whatman No. 1 filter and through 0.45 µm polyamide syringe filters (Chromafil Xtra, Macherey–Nagel, Germany). Sterilization of nC_60_ solution was performed in laminar flow hood (Airstream AC2-4E1, Esco, Singapur) using 0.45 µm polyamide syringe filters (Chromafil Xtra, Macherey–Nagel, Germany).

Characterization of nC_60_ solution included determination of particle size distribution and zeta potential. Dynamic light scattering (DLS) was used for determination of hydrodynamic size, and electrophoretic light scattering (ELS) for measurements of surface charge (zeta potential) of analysed samples. The measurements were conducted on a Zetasizer Nano ZS instrument (Malvern Instruments, UK). All DLS measurements were performed in triplicate, at 633 nm wavelength and a measurement angle of 173° (*backscatter detection*) at room temperature. The zeta potential measurements were conducted in duplicate.

### *Aspergillus flavus* growth and aflatoxin production in culture media

Suspension of *A. flavus* NRRL 3251 conidia preparation, inoculation as well as mycelia growth in aflatoxin-inducing YES medium were conducted as described by Kovač et al. (2017). Incubation was conducted in the dark at 29 °C, conditions which favour aflatoxin production (Yu 2012), for 168 h on a rotary shaker (KS 260 basic, IKA, Germany) set to 200 rpm in the presence of environmentally plausible nC_60_ concentrations (0, 10, 50 and 100 ng/mL). Samples were collected every 24 h from 48 to 168 h, following separation from YES media by filtration. Mycelia obtained at the same time-point were pooled and homogenised using pestle and mortar. The main part of homogenised mycelia was stored at – 80 °C until analysis of cell oxidative status, while 200 mg was dried until constant mass (24 h at 105 °C) to determine dry mycelial weight.

Quantitative analysis of aflatoxin content in culture filtrates was performed by a “dilute and shoot” method as described by Kovač et al. (2017). Recovery was assessed by spiking blank YES medium with aflatoxin standard solution at a concentration of 10 ng/mL, and it was 92% for aflatoxin B1. Instrumental limits of detection were 0.15 ng/mL, and limits of quantification were 0.5 ng/mL for all aflatoxins. All quantified aflatoxin concentrations were corrected for recovery.

### Disintegration of *A. flavus* mycelia

Extracts of *A. flavus* mycelia used for analysis of cell oxidative status were prepared by glass bead homogenization using a Bead Bug Microtube homogenizer (Benchmark Scientific, USA). Disintegration mixture contained 0.1 g of mycelia, 1 g of precooled, acid washed glass beads (diameter 0.5 mm) (Sigma Aldrich, Germany) and 1 mL of ice cold extraction buffer. Disintegration was performed at 4000 rpm in three cycles consisting of 2 min of disruption and 2 min of sample cooling on ice. Extracts were clarified by centrifugation at 15000×*g* and 4 °C for 20 min (Heraeus, Germany), and immediately used for analysis. Depending on the type of analysis, extraction buffers slightly differed in composition. Extracts used for antioxidant enzyme assays were prepared using 50 mM potassium phosphate buffer (pH 7.0) containing 1 mM EDTA-2Na, while buffers used in TBARS and GSH and GSSG assays additionally contained TCA (100 and 50 mg/mL, respectively).

### Oxidative status of *A. flavus* NRRL 3251

The non-enzymatic (TBARS concentration and GSH/GSSG ratio) and enzymatic ROS-dependent markers (Cu,Zn-SOD, Mn-SOD, catalase (CAT), glutathione peroxidase (GPX) and GR) of oxidative status were determined.

TBARS assay was performed according to Luschak and Gospodaryov (2005). TBARS concentration in mycelia extracts was evaluated spectrophotometrically (Helios γ, ThermoSpectronic, UK) at 535 nm and molar extinction coefficient of malondialdehyde (ε_535 nm_ = 156 × 103 L/cm/mol) was used for calculation. GSH and GSSG concentrations were estimated spectrofluorometrically (Cary Eclipse, Varian, Australia) according to Senft et al. (2000) using fluorescence indicator OPA.

Xanthine/xanthine oxidase/NBT assay (Angelova et al. 2005) was used for estimation of superoxide dismutase (EC 1.15.1.1) activities at 505 nm. Activities of cyanide sensitive Cu,Zn-SOD and the cyanide resistant Mn-SOD isoenzyme were also estimated. Total SOD activity was measured without, while Mn-SOD in the presence of mM potassium cyanide. Cu,Zn-SOD activity was calculated by substracting Mn-SOD from total SOD activity. CAT (EC 1.11.1.6) and GR (EC 1.8.1.7) activities were measured using spectrophotometric methods described by Reverberi et al. (2005), while GPX (EC 1.11.1.9) activity was measured at 340 nm according to Esworthy et al. (2005).

### Protein measurement

The Bradford assay was used to determine protein concentration in prepared extracts (Bradford 1976).

### Statistical analysis

Data presented in this paper are expressed as the mean value ± SEM from three separate experiments. The datasets were pooled and checked for normality of distribution by Shapiro–Wilk test and compared by nonparametric statistics methods (Friedman ANOVA and Kendall coefficient of concordance; Kruskal–Wallis ANOVA). The programme package Statistica 12.0 (Dell, USA) was used and differences were considered significant when the *p* value was < 0.05.

## Results

### Characterisation of nC_60_

Most of the nC_60_ nanoparticles in suspension were within 50–190 nm in diameter, and 21% had a hydrodynamic radius of 78 nm (Additional file 1: Figure S1a). Mean zeta potential value of the analysed nC_60_ solution was − 24.5 mV (Additional file 1: Figure S1b). These results of nC_60_ characterization are in accordance with a study conducted by Lyon et al. (2006) who reported the diameter of nC_60_ ranging from 30 to 100 nm. Also, 62.5% of nC_60_ nanoparticles in the prepared sample had diameters below 100 nm, which is in accordance with the EC Recommendation for a definition of the term “nanomaterial” (Rauscher et al. 2015).

### nC60 influence on mycelial growth, oxidative stress and aflatoxin production

**Fig. 1:**
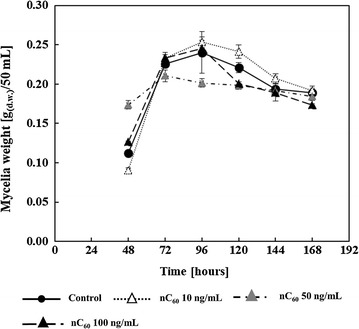
Influence of fullerene C_60_ nanoparticles (nC_60_) on *A. flavus* mycelial growth (expressed as gram of dry weight (g.d.w.) per 50 mL) in YES medium during 168 h at 29 °C. Data are represented as the mean ± SEM from three separate experiments

The effect of fullerene nanoparticles on *A. flavus* growth is presented in Fig. 1. A measurable mycelium mass was reached after 48 h of incubation. Collectively, only 50 ng/mL nC_60_ affected mycelial growth, causing a statistically significant (H = 32.04; p = 0.0016) higher dry mycelial mass after 48 h of growth (by 54%), but also a slight reduction in dry mycelium weight during exponential growth phase (after 72 h) and between 96 and 120 h of stationary growth phase. These statistically significant mass reductions were in a range from 6 to 16%. On the other hand, mycelial dry weight after 120 h of growth was similar to control as well as to samples treated with 10 and 100 ng/mL concentration of nC_60_. Both control and nC_60_-treated samples’ mycelial biomass showed a decreasing trend after 120 h of growth.

**Fig. 2:**
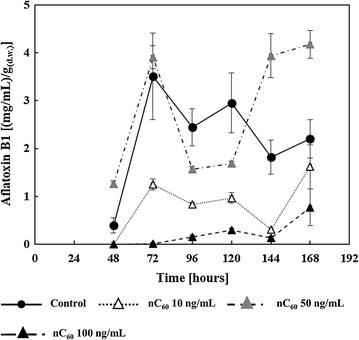
Influence on fullerene C_60_ nanoparticles (nC_60_) on *A. flavus* aflatoxin B1 production in the culture filtrates during 168 h of incubation at 29 °C. Aflatoxin content is expressed in ng aflatoxin B1 per g mycelial dry weight and represented as the mean ± SEM from three separate experiments

Analysis of control and nC_60_-treated samples on aflatoxin presence revealed dominance of aflatoxin B1 in growth media, while aflatoxin B2 levels were below LOQ and aflatoxin G1 and G2 were below LOD. Accordingly, only aflatoxin B1 levels are presented in Fig. 2. Fullerene nanoparticles caused a strong antiaflatoxigenic effect at all time-points at concentrations of 10 and 100 ng/mL, whereas no aflatoxin B1 was detected in the media up to 48 h (10 ng/mL) and 72 h (100 ng/mL) of growth. Aflatoxin B1 production was reduced by an average 67% for 10 ng/mL concentration and by an average 90% for 100 ng/mL concentration of nC_60_. The medium nC_60_ concentration of 50 ng/mL slightly decreased aflatoxin B1 production only between 96 and 120 h of incubation period, while further incubation resulted in approximately twofold higher levels compared to controls.

**Fig. 3:**
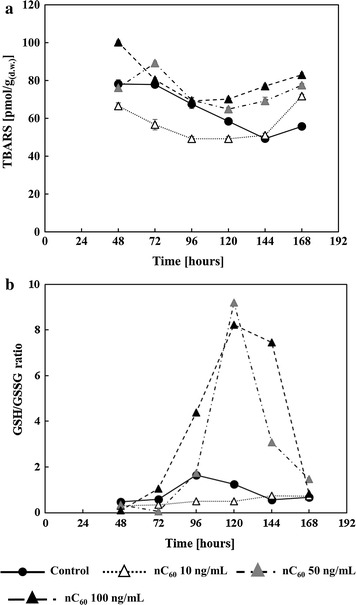
Influence of fullerene C_60_ nanoparticles (nC_60_) on: **a** lipid peroxidation and **b** reduced and oxidised glutathione ratio (GSH/GSSG) in *A. flavus* mycelia during 168 h growth period in YES medium at 29 °C. Lipid peroxides are expressed as pmols of thiobarbituric acid reactive substances (TBARS) per dry weight of mycelia and represented as the mean ± SEM from three separate experiments

**Fig. 4:**
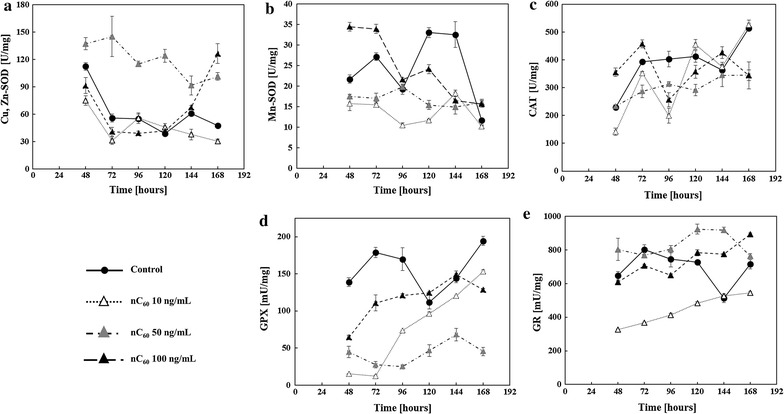
Modulation of antioxidant enzyme activities in *A. flavus* mycelium by fullerene C_60_ nanoparticles (nC_60_) during 168 h growth period in YES medium at 29 °C. Enzyme activity is expressed in U/mg of protein and represented as the mean ± SEM from three separate experiments. Enzymes: **a** copper, zinc superoxide dismutase (Cu,Zn-SOD), **b** manganese superoxid dismutase (Mn-SOD), **c** catalase (CAT), **d** glutathione peroxidase (GPX), and **e** glutathione reductase (GR)

Since, aflatoxin B1 production is a secondary defence mechanism of fungi against oxidative stress in the cell (Reverberi et al. 2012) ROS-dependent oxidative stress biomarkers TBARS (Fig. 3a), GSH/GSSG (Fig. 3b) and activities of antioxidative enzymes (Fig. 4a–e) were determined.

The findings suggest that TBARS responded to nC_60_ treatments (Fig. 3a). Both 50 and 100 ng/mL nC_60_ led to somewhat elevated TBARS levels, especially during the final hours of incubation. The lowest dose had the opposite effect. In agreement with the TBARS results, the GSH/GSSG ratio (Fig. 3b) did not change significantly when treated with 10 ng/mL nC_60_. On the other hand, the GSH/GSSG ratio increased dramatically after 72 h of incubation with 50 and 100 ng/mL. GR activity displayed a similar pattern of responses to nC_60_ treatments (Fig. 4e).

Cu,Zn-SOD activity was found to be affected and increased only in the presence of 50 ng/mL nC_60_ (Fig. 4a), while Mn-SOD (Fig. 4b), CAT (Fig. 4c), and GPX activities (Fig. 4d) were decreased at almost all time points during incubation with all tested nC_60_ concentrations.

## Discussion

The nC_60_ suspensions were prepared by long lasting stirring (30 days) of C_60_ powder in ultrapure water, as described above, in order to obtain environmentally most relevant nC_60_ form with no solvent or solubilizing agents involved. In this way, all residual solvent or solubilizing agent effects on observed nC_60_ interaction with *A. flavus* were excluded, and a potential scenario of C_60_ accidental release or disposal in the environment was mimicked in controlled laboratory conditions (Lyon et al. 2008; Lyon and Alvarez 2008). Noted minor differences in mycelial growth between treatments notwithstanding (Fig. 1), the overall impression is that this range of nC_60_ levels did not affect *A. flavus* growth substantially in either direction. This is in agreement with previous observations on eukaryotic microorganisms (Aoshima et al. 2009; Hadduck et al. 2010).

Quantification of aflatoxin B1 concentrations (Fig. 2 suggested general suppression of aflatoxin synthesis in the presence of nC_60_. Explaining the deviating results at the middle, 50 ng/mL nC_60_ level, is difficult but it could involve the distinct effects of C_60_ depending on concentration, medium, target organism, etc., as reported by other authors. For instance, the antioxidant effects of fullerene nanoparticles in rats were reported by Gharbi et al. (2005) and Baati et al. (2012). However, both in vitro (Isakovic 2006b) and in vivo (Oberdörster 2004) studies also determined prooxidative, ROS-related effects of fullerene nanoparticles. Moreover, Lyon et al. (2008) and Lyon and Alvarez (2008) demonstrated ROS-independent oxidative damage of bacterial cells by fullerene nanoparticles. Taking into account the relationship between *A. flavus* redox status and aflatoxin synthesis, and the versatility of nC_60_ action, an initial interpretation would assume antioxidative effects of nC_60_ at all tested levels except 50 ng/mL after 144 h of incubation.

Aflatoxin production is a secondary defence mechanism of fungi against oxidative stress in the cell (Reverberi et al. 2012; Hong et al. 2013; Grintzalis et al. 2014). Therefore, oxidative status of *A. flavus* cells was evaluated by determining the hallmarks of cellular ROS-inflicted damage and their elimination (Reverberi et al. 2005; Narasaiah et al. 2006): TBARS (Fig. 3a), GSH/GSSG ratio (Fig. 3b) and activities of antioxidative enzymes (Fig. 4).

The TBARS usually result from damage to lipids by hydroxyl radical which might be implicated in aflatoxin B1 production (Grintzalis et al. 2014). The present results on TBARS content (Fig. 3a) pointed to antioxidative action of C_60_ nanoparticles at the lowest concentration. The antioxidative effect was also observed in all other monitored oxidative status parameters (Figs. 2, 3, 4) in the presence of 10 ng/mL of nC_60_. Treatments with higher doses (50 and 100 ng/mL) exerted prooxidative effects in the fungus, leading to an increased formation of TBARS (Fig. 3a), and the GSH/GSSG ratio (Fig. 3b) which indicated an immense upregulation of GSH synthesis with the purpose of antioxidative defence. A congruous pattern of activity of GR, an enzyme involved in GSH maintenance in the cell (Li et al. 2009), was recorded for the selected range of nC_60_ doses (Fig. 4e).

An increase of Cu,Zn-SOD activity points to prooxidative effects of nC_60_, but curiously, only when *A. flavus* was treated with 50 ng/mL (Fig. 4a). Activity remained at control levels with the highest dose. Upregulation of Cu,Zn-SOD was reported to be a result of adaptive response of filamentous fungi to elevated ROS levels produced by a superoxide anion generator (Angelova et al. 2005). Presumably, nC_60_ generated superoxide anion, and such generation may have intensified in the YES medium which contains reducing agents as NADH or NADPH (Yamakoshi et al. 2003; Badireddy et al. 2007).

Since 100 ng/mL nC_60_ displayed prooxidative effects in terms of increased TBARS, GSH and GR, the absence of its influence on Cu,Zn-SOD activity may be an indication of cytotoxic effects of fullerene nanoparticles. The toxic effect of nC_60_ could be related to an interaction with cell membrane (Chen et al. 2010), absorption of nC_60_ and distribution in the organelles (Isaacson et al. 2009), etc. Additionally, inhibition of microtubule formation, mitochondrial dysfunction, disturbance of membrane transport, signal transduction, etc. (Johnson-Lyles et al. 2010; Ratnikova et al. 2011; Grebowski et al. 2013) reported for fullerenol nanoparticles should be considered. According to Chae et al. (2010), increased water solubility of fullerene nanoparticles results from surface hydroxylation. Thus, it seems quite possible that a part of the smallest fullerene nanoparticles acted as fullerol nanoparticles, whose effects on *A. flavus* were described by Kovač et al. (2017).

Fullerene nanoparticles could have, for example, either suppressed aflatoxin biosynthesis or interfered with aflatoxin release in the media (Holmes et al. 2008). In fact, the cytotoxic effects occurring at 100 ng/mL could reconcile prooxidative effects of nC_60_ (Fig. 3) with the lower concentration of aflatoxins in the growth media (Fig. 2).

The reduced activities of Mn-SOD, CAT and GPX (Fig. 4b–d) may be ascribed to antioxidative action of nC_60_, as mentioned above for the lowest (10 ng/mL) dose, to the inhibition of enzymes and cytotoxic effects, and/or the levels of peroxides were not large enough to upregulate CAT and GPX.

In conclusion, the present results clearly demonstrated volatility of nC_60_ effect on the mycotoxigenic potential of *A. flavus*. It may stimulate aflatoxin synthesis due to prooxidative action on the fungal cell, as detected with 50 ng/mL. An antiaflatoxigenic effect was observed at both 10–100 ng/mL nC_60_, with disparate mechanisms of action. An apparent antioxidative effect of 10 ng/mL nC_60_ reduced biosynthesis of aflatoxins (Reverberi et al. 2005), while 100 ng/mL nC_60_ exerted both prooxidative (measured) and cytotoxic (assumed) effects in fungal cells, thus effectively shutting down aflatoxin biosynthesis and/or their release from the cell. Further studies should extend the range of tested nC_60_ levels, investigate behaviour of photosensitised fullerene, and response of mycotoxigenic fungi in environmental conditions which are expected to favour aflatoxin synthesis due to climate change (Battilani et al. 2016; Medina et al. 2017).

## Additional file

**Additional file 1.** Size distributions by number (a) and zeta potential (b) of fullerene C60 nanoparticles.

## Abbreviations

nC_60_: nanoparticles of fullerene C_60_
ROS: reactive oxygen species
GR: glutathione reductase
XOD: xanthine oxidase
NBT: nitrotetrazolium blue chloride
EDTA-4Na: ethylenediaminetetraacetic acid tetrasodium salt
GSH: glutathione reduced
GSSG: glutathione oxidized
OPA: *o*-phthalaldehyde
NADPH: β-nicotinamide adenine dinucleotide 2′-phosphate reduced tetrasodium salt
NEM: *N*-ethylmaleimide
DT: sodium dithionite
H_2_O_2_: hydrogen peroxide
TCA: trichloroacetic acid
EDTA-2Na: ethylenediaminetetraacetic acid disodium salt
TBA: 2-thiobarbituric acid
YES: yeast extract sucrose
TBARS: thiobarbituric acid reactive substances
SOD: superoxide dismutase
Cu,Zn-SOD: copper,zinc superoxide dismutase
Mn-SOD: manganese superoxide dismutase
GPX: glutathione peroxidase
CAT: catalase
