# Supplementary material for: Antiaflatoxigenic effect of fullerene C60 nanoparticles at environmentally plausible concentrations
Source: AMB Express. 2018 Feb 5;8:14. doi: 10.1186/s13568-018-0544-0 (PMC5799089; doi:10.1186/s13568-018-0544-0)
Supplement: Supplementary file 1 — Additional file 1. Size distributions by number (a) and zeta potential (b) of fullerene C60 nanoparticles. [file 13568_2018_544_MOESM1_ESM.pdf]

AMB Express

ANTIAFLATOXIGENIC EFFECT OF FULLERENE C_{60} NANOPARTICLES AT ENVIRONMENTALY PLAUSIBLE CONCENTRATIONS

Tihomir Kovač[a]*, Bojan Šarkanj[a], Tomislav Klapec[a], Ivana Borišev[b], Marija Kovač[c], Ante Nevistić[c], Ivica Strelec[a]

[a] Josip Juraj Strossmayer University of Osijek, Faculty of Food Technology, Department of Applied Chemistry and Ecology, Franje Kuhača 20, 31000 Osijek, Croatia

[b] University of Novi Sad, Faculty of Sciences, Department of Chemistry, Biochemistry and Environmental protection, Trg Dositeja Obradovića 3, 21000 Novi Sad, Serbia

[c] Inspecto d.o.o., Električne centrale 1, 31400 Đakovo, Croatia

*Corresponding Author

Phone: +385 31 224 378; fax: +385 31 207 115; e-mail: tihomir.kovac@ptfos.hr

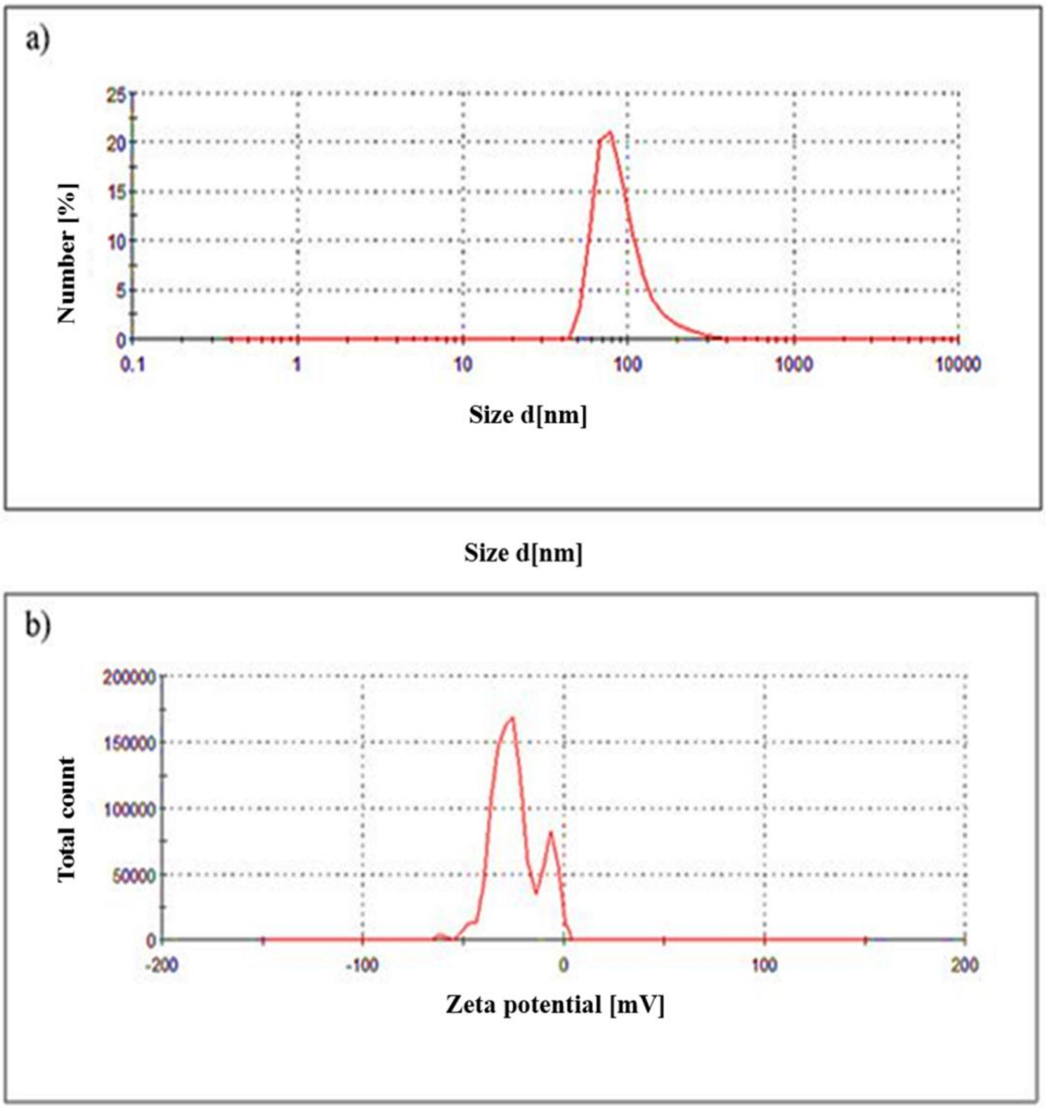

Figure S1. Size distributions by number (a) and zeta potential (b) of fullerene C_{60} nanoparticles.
